# Genetic diversity of the Sudanese: insights on origin and implications for health

**DOI:** 10.1093/hmg/ddab028

**Published:** 2021-04-16

**Authors:** Muntaser E Ibrahim

**Affiliations:** Institute of Endemic Diseases, University of Khartoum, Khartoum, Sudan

## Abstract

By virtue of their cultural, linguistic and genetic legacies, many populations from Sudan have deep histories in the region and retain high genetic diversities. Sudan’s location in north east Africa, a unique spot believed to act as a climatic refuge during periods of climate extremes, might have dictated that fate. Among the marked consequences of this diversity is the potential to provide information on the origin and structure of human populations within and outside the continent, as well as migration patterns towards various parts of the African continent, and out of Africa. The diverse Sudanese gene pool further has the potential to inform on genetic adaptations driven by culture and the environment resulting in unique and interesting traits, some of which are yet to be investigated. In addition, these genomes could offer clues to complex issues of causation amidst the challenge of new paradigms in biology underpinned by the genomic revolution.

Populations from Sudan contribute to inferences about the deep history of humans within Africa. Humans were new comers to a 5 billion years old world, which was already endowed with the gift of life for at least 3.5 billion years. The estimated naissance of our sub-species is in the mere range of 100–300 thousand years ago (kya) (1,2). Several lines of evidence from the paleontological field highlight the central role of East Africa in deep human history (1,3,4). Traversed by the Nile and other running and seasonal rivers and bordering the Sahara, the Savannah and the Ethiopian plateau, all contribute to the unique setting that forms northeast Africa, where the highest abundance of hominin fossils exist. The reason is simple: its location. Northeast Africa and its extension to the Sahara form a natural enclave during times of extreme environmental conditions and the succession of climatic cycles in what is known as climate pulses (4). During the last Glacial–Interglacial transition, known as the African Humid Period, the Sahara was not a desert. Rock drawings depict scenery of greenery and a lush Savannah environment with wildlife similar to the east African Serengeti today. It was the ideal incubator and likely the inspiring environment that led to the changes that were about to take place.

Although the location in Africa where humans emerged cannot be traced to an exact coordinate, substantial inferences can be made based on both genetics and archeology, where abundant evidence supports the important role played by northeast Africa (5,6). Early language forms (7), early phenotypic traits and early adaptive traits may have risen as a response to the challenges created by the spectrum of environments encountered by early humans. The axis of the Sahel that extends longitudinally from the plain to the mountain, from dry to wet, and cold to hot, presents a spectrum rarely found elsewhere. With evidence for the continuity of human settlement between the Red Sea and Lake Chad, Sudan and the region extending around is characterized by abundant lithic artifacts and fossil human remains through the main periods of modern human evolution from the Middle Stone Age (300–30 kya), a period which probably does not extend back further than 190 000 years ago (8), to the Mesolithic (120–6 kya). During the latter, known as the African Humid Period (AHP), a fundamental shift in climate conditions to a moist environment occurred from ~12 kya, giving rise on both sides of the Nile Valley to a grassland. The ‘Wadi Hawar’ in northwest Sudan became a succession of rivers and lakes, allowing human habitation in what are now desert zones, partially in stable settlements along river courses and partially in seasonally occupied areas. Fishing, hunting and gathering of plants and fruits were primary sources of survival (9). Around the Sudanese capital Khartoum, the remains of one of the very first hunting villages to be associated with fishery and pottery have been found. It is one of the earliest sites with pottery in Africa. The Neolithic Revolution (8000–4500 BP) is the term given to the first agricultural revolution, describing the transition from hunting and gathering to pastoralism and farming. As first adopted by various independent prehistoric human societies, the Neolithic era in Sudan was located between the south of Egypt and central Sudan around the Nile Valley. The second half of the sixth millennium bc is distinguished by an increasingly dry climate. The origins and groupings of the population are difficult to reconstruct, since few well-preserved skeletal remains have been discovered (10).

Partial genetic evidence also suggests an extended period of human settlement before the first major expansion out of Africa took place around 40–60 000 years ago although the date of the main ‘out of Africa’ dispersal remains vague and with wide margins of error. Scientists believe expansions might have been the outcome of a combination of reasons including biological change that led to the appearance of new traits and cultures including the evolution of arts, language and new tool making technologies. Human long range migration likely did not occur first during the out of Africa scenario. Demographic expansions of hunter gatherers might have occurred earlier. Genetic studies, based on both modern-day hunter gatherers and ancient DNA, show a cline of genetic relatedness between eastern and southern African hunter gatherers (11). Traces of click languages among hunter gatherers may be a remaining legacy of population contact and genetic interchange along this route (12,13). Cultural changes reflected in new tools led subsequently to the advent of agriculture and animal domestication, the latter of which was the driving force of yet further demographic expansions.

Migratory routes might have followed the course of rivers in the eastern parts of the current Sahara, like the Wadi Hawar, which has been running until recently, since availability of water was a crucial element in human survival during migratory movements. Dispersal in environmental range is an integral behavior of many biological species, but the pace and the striking adaptability accompanying movement is what characterizes human expansions. Evidence for early expansions of east Africans predating the main expansion involving the rest of world population is conspicuous in the network analysis by Elhassan *et al.* (14).

Migrations affect not only physical placement and replacement of biological entities in time and space; it is a potent mechanism of evolutionary dynamics, of introgression, genetic drift, selection and other genetic effects. Although cases of wide range expansion and isolation existed in Africa, for instance, that of, the Bantu-speaking agro-pastoralists, the Afro-Asiatic pastoralists and the Nilotic expansions (15–17), emphasis continues to be placed on the out of Africa episodes (18,19) despite the fact that humanity spent most of its biological history within the African continent (17). When seeking markers of ancestry, early studies chose either mitochondrial DNA or Y chromosome markers, as they are both spared the shuffling impact of recombination. In Sudan, the mitochondrial gene pool of the maternal lineages is likely to have been shaped by a longer history of in situ evolution (20), while the Y chromosome despite its antiquity is the one that mirrors population linguistic and geographic structures as well as recent migrations within the past 300–400 years (21,22).

Further benefits arise from studies defining population structure and disease associations by using haplotype analysis and SNP tagging approaches. It has been shown that it is possible with a limited set of population markers to reveal hidden structures within seemingly homogenous populations. For example, the sickle cell mutation is found on different haplotypes among ethnic groups in Sudan (23), and analyses related to population structure suggest that this mutation was only recently introduced into the eastern Sahel (22). Further analysis on malaria susceptibility loci in the Hausa, Massalit and Sinnar revealed further population-specific associations (24). Another study on ethnic differences for visceral leishmaniasis in Sudan used a genome-wide scan to reveal associations with markers on chromosomes 1 and 6 (25). More recently, genetic association was used to explore the association of SARS-CoV-2 infection and death phenotypes with a particular Y chromosome haplotype (26). These approaches can also be used to date the introduction and ancestral state of ambiguous loci in different populations based on the contrasting genotype and allele frequency patterns between Africans and non-Africans (27).

## Population Structure, Culture and Differentiation

The term Sudan was used until recently in reference to geographical stretches along the area of the Sahel from the Red Sea to the Atlantic. Multiple sets of genetic evidence attest to the deep history of certain populations in this area (28), and their association with early cultural revolutions (29).

Genetic drift and selection are the most potent evolutionary forces; however, populations may also be set apart by cultural barriers and hence population structure, which is rooted in many complex cultural features including language barriers (30) and consanguinity, a custom still widely practiced in Northern Africa and the Sahel. In Sudan, consanguinity has been associated with genetic disorders particularly those of an autosomal recessive nature (31). A spectacular manifestation of the impact of culture on disease burden and accumulation of deleterious mutations is the elevated frequency of the sickle cell mutation (HBS) among the ‘Bagara’ of western Sudan. The frequency is among the highest in Sudan and Africa apparently due to consanguinity (22). Bagara are nomadic pastoralists of Arab descent who carrying predominantly the J1 Y chromosome haplotypes in their male lineages. Their migration and admixture within the present Sudan from the area around Lake Chad is estimated to have occurred during the past 4 millennia. It is believed that they introduced HBS mutation with its unique diversity of typical and atypical haplotypes (23) to the local Nilo-Saharan populations who rarely practice cultures of internal marriages and with much lower frequency of carriers and disease.

East African genomes and those from Sudanese populations in particular could provide significant insights into the possible biological processes that shaped the adaptations within our species, particularly during the Holocene when the human genome became increasingly influenced by human’s own cultural transformations of agriculture and pastoralist practices. The fact that the most diverse set of variants associated with the lactase persistence trait, influencing the expression of the enzyme responsible for the digestion of lactose, the main sugar in milk, are present within east African populations including Sudanese is a testimony to the impact of cultural practices in the region (32). Previously neutral and random mutations circulating in these populations likely became more abundant where the environment favored their selection (33). The Beja and Fulani for example are both endowed with a number of lactase persistence (LP) mutations that are consistent with the tolerance of dairy consumption in adults (34). Rock drawings, carvings on ancient temples, burial rites and customs support a rich history of association with cattle, raising some fundamental questions about the history of cattle domestication and the spread of a pastoralist culture (32). The striking association between the Y-chromosome haplogroup E, pastoralist culture and the Afro-Asiatic family of languages has been suggested to have sprouted from a common culture that flourished in the Sahara during the AHP (15).

Genetic adaptation in the context of cultural practices can also inform evolutionary dynamics and provide insights through analysis of allele frequency patterns for classes of adaptive traits, specifically when examining genes in genomic regions within human populations that have diverged culturally. Given the differences between the life styles of our ancestors and by examining the genomic architecture of hunter-gatherers (35), we can compare genetic loci that are thought to influence the various physiological mechanisms that underlie the development of some traits associated with adaptive novelties. These include cardiovascular diseases and measuring the potential effect of such mutations on resting blood pressure (36).

## Implications of Diversity for Health and Complex Inheritance

The current resurgence of pandemics and global climatic and behavioral changes are bound to reflect in our health, raising concerns about how Africans are genomically equipped to deal with such changes. Despite the stark under-representation of Africans in genomics databases (37), that paucity is contrasted, ironically, by the pronounced African genetic diversity and the marked evidence for biological, environmental and climate-related adaptation (38) within the African genomes.

Genomes of east Africans, and Sudanese in particular, are crucial to consider for the following reason: mounting evidence exists that many human adaptive traits were acquired in Africa, where the modern human species evolved. Many of those adaptive traits like skin color and other physical and physiological traits are products of a deeper African evolutionary history, including natural selection (39). They assume importance not only because of their ancestral state and antiquity but for their large effective population size. The effective population size, a term that describes the amount of genetic variation contributing to the founding of a population, denotes also its level of genetic diversity. The effective human founding population size (*N_e_*) is estimated to be between 1000 and 10 000 individuals—this number is believed to have fluctuated over time, often suffering severe bottlenecks (16,40).

*N_e_*, an expression of the amount of unique variations or number of mutations in a particular group, is quite pertinent to the practice of genomics in Africa. The significance of population size being a measure of genetic diversity and fitness is multiple, pertinent to most of the challenges we face as a species, our health and social being, and our relationship with the environment. The larger the effective size of a species, the better its chances of confronting adversities and survival.

There is no better example to justify understanding the repercussion of a high genetic diversity and high effective population size than cancer biology. Cancer as a disease is the ultimate embodiment of a complex phenomenon. Conventionally, cancer genetics has focused on mutational events that have their primary effects on the cell. Recently, however, that focus has widened, with evidence for the importance of epigenetic occurrences and cellular interactions in cancer development. Differential methylation analysis corroborated these results revealing the epigenetic dysregulation of major developmental pathways including the Hippo signaling pathway (41). In Sudan, the peculiarity of cancer etiology was manifested in instances where the genetic predisposition of breast cancer did not conform to known BRCA mutations and where the role EBV virus was implicated in Sudan but not in Eritrea (42,43).

The predicament of variant interpretation is accentuated further as conventional variant calling methods of pathogenic alleles in exome and genome sequencing requires the presence of the non-pathogenic alleles as genome references alleles. African data have illustrated that some reference alleles may be pathogenic and should be treated with caution. Under conditions of heterogeneity of allele frequency, it was proven problematic leading to the possible miss-interpretation and oversight of homozygous disease variants (44).

In brief, without understanding the intricacies of the regulation of biological networks and how genes/proteins interact, neither the genetic differences nor the effects of selection will be fully understood and properly elucidated (15). Africa, being the largest reservoir of human genetic variation, and populations of the Sudan and eastern Africa will be confronting bigger challenges in genomics research and practices, particularly the future pursuit of individualized medicine.

It is apparent that detailed analysis and understanding of human population origins and diversity as reflected in extant populations may provide useful insights pertinent to human health and the challenges outlined above, rendering systematic and coordinated analysis of these genomic legacies both relevant and imperative.
